# Supplementary material for: Privacy concerns regarding personal health information in Myanmar: A cross-sectional survey in a least developed country
Source: PLOS Digit Health. 2026 Mar 26;5(3):e0001007. doi: 10.1371/journal.pdig.0001007 (PMC13020815; doi:10.1371/journal.pdig.0001007)
Supplement: S1 Table — (DOCX) [file pdig.0001007.s002.docx]

Table S1. Associations between antecedent factors and PHIPC.

|  | Low Concerns^a^  (n=94) | High Concerns^a^  (n=330) | Crude OR (95% CI) | Adjusted OR (95% CI) |
| --- | --- | --- | --- | --- |
|  |  |  |  |  |
| **Age Group** |  |  |  |  |
| 18-24 years | 9 (12.16) | 65 (87.84) | Reference | Reference |
| 25-31 years | 39 (20.63) | 150 (79.37) | 0.53 (0.23, 1.12) | 0.56 (0.20, 1.48) |
| 32-38 years | 24 (26.97) | 65 (73.03) | 0.38 (0.15, 0.84)* | 0.39 (0.13, 1.08) |
| 39-45 years | 5 (14.29) | 30 (85.71) | 0.83 (0.26, 2.90) | 1.23 (0.33, 5.05) |
| >45 years | 17 (45.95) | 20 (54.05) | 0.16 (0.06, 0.41)*** | 0.29 (0.09, 0.88)* |
| **Sex** |  |  |  |  |
| Female | 64 (25.5) | 187 (74.5) | Reference | Reference |
| Male | 27 (15.98) | 142 (84.02) | 1.80 (1.10, 3.00)* | 1.57 (0.89, 2.80) |
| Prefer not to say | 3 (75) | 1 (25) | 0.11 (0.01, 0.91) | 0.33 (0.01, 3.01) |
| **Residence** |  |  |  |  |
| Rural | 20 (19.23) | 84 (80.77) | Reference | Reference |
| Urban | 74 (23.12) | 246 (76.88) | 0.79 (0.45, 1.35) | 0.97 (0.45, 2.03) |
| **Education** |  |  |  |  |
| Bachelor’s degree or higher | 60 (23.17) | 199 (76.83) | Reference | Reference |
| Lower than bachelor’s degree | 34 (20.61) | 131 (79.39) | 1.16 (0.73, 1.88) | 0.96 (0.46, 2.06) |
| **Occupation** |  |  |  |  |
| Healthcare | 34 (29.06) | 83 (70.94) | Reference | Reference |
| Non-healthcare | 49 (20.5) | 190 (79.5) | 1.59 (0.95, 2.63) | 1.70 (0.87, 3.39) |
| Unemployed | 11 (16.18) | 57 (83.82) | 2.12 (1.02, 4.71) | 1.73 (0.63, 4.99) |
| **EMR Awareness** |  |  |  |  |
| Have not heard | 15 (16.3) | 77 (83.7) | Reference | Reference |
| Have heard, but not understand | 39 (27.08) | 105 (72.92) | 0.52 (0.26, 1.00) | 0.67 (0.30, 1.49) |
| Understand | 40 (21.28) | 148 (78.72) | 0.72 (0.37, 1.36) | 1.34 (0.52, 3.43) |
| **Perceived Health Status** |  |  |  |  |
| Poor | 21 (51.22) | 20 (48.78) | Reference | Reference |
| Well | 73 (19.06) | 310 (80.94) | 4.46 (2.29, 8.71)*** | 3.72 (1.69, 8.23)** |
| **Health Concerns** |  |  |  |  |
| Not concerned | 71 (39.23) | 110 (60.77) | Reference | Reference |
| Concerned | 23 (9.47) | 220 (90.53) | 6.17 (3.71, 10.6)*** | 5.26 (3.02, 9.45)*** |

Table S2. Associations between antecedent factors and PHIPC for collection.

|  | Low Concerns^a^  (n=320) | High Concerns^a^  (n=104) | Crude OR (95% CI) | Adjusted OR (95% CI) |
| --- | --- | --- | --- | --- |
|  |  |  |  |  |
| **Age Group** |  |  |  |  |
| 18-24 years | 51 (68.92) | 23 (31.08) | Reference | Reference |
| 25-31 years | 145 (76.72) | 44 (23.28) | 0.67 (0.37, 1.23) | 0.69 (0.31, 1.51) |
| 32-38 years | 72 (80.9) | 17 (19.1) | 0.52 (0.25, 1.07) | 0.55 (0.23, 1.28) |
| 39-45 years | 25 (71.43) | 10 (28.57) | 0.89 (0.36, 2.11) | 0.91 (0.33, 2.42) |
| >45 years | 27 (72.97) | 10 (27.03) | 0.82 (0.33, 1.94) | 0.62 (0.23, 1.62) |
| **Sex** |  |  |  |  |
| Female | 184 (73.31) | 67 (26.69) | Reference | Reference |
| Male | 132 (78.11) | 37 (21.89) | 0.77 (0.48, 1.21) | 0.90 (0.55, 1.46) |
| Prefer not to say | 4 (100) | 0 (0) | 0.00 | 0.00 |
| **Residence** |  |  |  |  |
| Rural | 84 (80.77) | 20 (19.23) | Reference | Reference |
| Urban | 236 (73.75) | 84 (26.25) | 1.49 (0.88, 2.64) | 1.53 (0.80, 3.03) |
| **Education** |  |  |  |  |
| Bachelor’s degree or higher | 198 (76.45) | 61 (23.55) | Reference | Reference |
| Lower than bachelor’s degree | 122 (73.94) | 43 (26.06) | 1.14 (0.73, 1.79) | 1.21 (0.61, 2.38) |
| **Occupation** |  |  |  |  |
| Healthcare | 88 (75.21) | 29 (24.79) | Reference | Reference |
| Non-healthcare | 182 (76.15) | 57 (23.85) | 0.95 (0.57, 1.60) | 1.01 (0.55, 1.87) |
| Unemployed | 50 (73.53) | 18 (26.47) | 1.09 (0.55, 2.15) | 1.15 (0.47, 2.78) |
| **EMR Awareness** |  |  |  |  |
| Have not heard | 71 (77.17) | 21 (22.83) | Reference | Reference |
| Have heard, but not understand | 106 (73.61) | 38 (26.39) | 1.21 (0.66, 2.26) | 1.19 (0.60, 2.39) |
| Understand | 143 (76.06) | 45 (23.94) | 1.06 (0.59, 1.95) | 1.09 (0.49, 2.48) |
| **Health Status** |  |  |  |  |
| Poor | 29 (70.73) | 12 (29.27) | Reference | Reference |
| Well | 291 (75.98) | 92 (24.02) | 0.76 (0.38, 1.61) | 0.78 (0.36, 1.75) |
| **Health Concerns** |  |  |  |  |
| Not concerned | 122 (67.4) | 59 (32.6) | Reference | Reference |
| Concerned | 198 (81.48) | 45 (18.52) | 0.47 (0.30, 0.73)*** | 0.46 (0.28, 0.74)** |

Table S3. Associations between antecedent factors and PHIPC for errors.

|  | Low Concerns^a^  (n=150) | High Concerns^a^  (n=274) | Crude OR (95% CI) | Adjusted OR (95% CI) |
| --- | --- | --- | --- | --- |
|  |  |  |  |  |
| **Age Group** |  |  |  |  |
| 18-24 years | 23 (31.08) | 51 (68.92) | Reference | Reference |
| 25-31 years | 63 (33.33) | 126 (66.67) | 0.90 (0.50, 1.59) | 0.67 (0.32, 1.39) |
| 32-38 years | 33 (37.08) | 56 (62.92) | 0.77 (0.40, 1.47) | 0.66 (0.30, 1.43) |
| 39-45 years | 13 (37.14) | 22 (62.86) | 0.76 (0.33, 1.80) | 0.73 (0.28, 1.91) |
| >45 years | 18 (48.65) | 19 (51.35) | 0.48 (0.21, 1.07) | 0.49 (0.20, 1.21) |
| **Sex** |  |  |  |  |
| Female | 94 (37.45) | 157 (62.55) | Reference | Reference |
| Male | 52 (30.77) | 117 (69.23) | 1.35 (0.89, 2.05) | 1.28 (0.82, 2.01) |
| Prefer not to say | 4 (100) | 0 (0) | 0.00 | 0.00 |
| **Residence** |  |  |  |  |
| Rural | 50 (48.08) | 54 (51.92) | Reference | Reference |
| Urban | 100 (31.25) | 220 (68.75) | 2.04 (1.30, 3.20)** | 2.30 (1.28, 4.17)** |
| **Education** |  |  |  |  |
| Bachelor’s degree or higher | 81 (31.27) | 178 (68.73) | Reference | Reference |
| Lower than bachelor’s degree | 69 (41.82) | 96 (58.18) | 0.63 (0.42, 0.95)* | 0.64 (0.34, 1.20) |
| **Occupation** |  |  |  |  |
| Healthcare | 45 (38.46) | 72 (61.54) | Reference | Reference |
| Non-healthcare | 80 (33.47) | 159 (66.53) | 1.24 (0.78, 1.96) | 1.68 (0.95, 2.99) |
| Unemployed | 25 (36.76) | 43 (63.24) | 1.07 (0.58, 2.01) | 1.55 (0.68, 3.61) |
| **EMR Awareness** |  |  |  |  |
| Have not heard | 29 (31.52) | 63 (68.48) | Reference | Reference |
| Have heard, but not understand | 63 (43.75) | 81 (56.25) | 0.59 (0.34, 1.02) | 0.40 (0.20, 0.76)** |
| Understand | 58 (30.85) | 130 (69.15) | 1.03 (0.60, 1.76) | 0.69 (0.31, 1.52) |
| **Health Status** |  |  |  |  |
| Poor | 20 (48.78) | 21 (51.22) | Reference | Reference |
| Well | 130 (33.94) | 253 (66.06) | 1.85 (0.96, 3.55) | 1.37 (0.65, 2.85) |
| **Health Concerns** |  |  |  |  |
| Not concerned | 71 (39.23) | 110 (60.77) | Reference | Reference |
| Concerned | 79 (32.51) | 164 (67.49) | 1.34 (0.90, 2.00) | 1.21 (0.77, 1.89) |

Table S4. Associations between antecedent factors and PHIPC for unauthorized secondary use.

|  | Low Concerns^a^  (n=105) | High Concerns^a^  (n=319) | Crude OR (95% CI) | Adjusted OR (95% CI) |
| --- | --- | --- | --- | --- |
|  |  |  |  |  |
| **Age Group** |  |  |  |  |
| 18-24 years | 11 (14.86) | 63 (85.14) | Reference | Reference |
| 25-31 years | 44 (23.28) | 145 (76.72) | 0.58 (0.27, 1.15) | 0.66 (0.25, 1.66) |
| 32-38 years | 25 (28.09) | 64 (71.91) | 0.45 (0.20, 0.96)* | 0.52 (0.19, 1.37) |
| 39-45 years | 6 (17.14) | 29 (82.86) | 0.84 (0.29, 2.65) | 1.32 (0.39, 4.77) |
| >45 years | 19 (51.35) | 18 (48.65) | 0.17 (0.06, 0.40)*** | 0.26 (0.09, 0.74)* |
| **Sex** |  |  |  |  |
| Female | 74 (29.48) | 177 (70.52) | Reference | Reference |
| Male | 28 (16.57) | 141 (83.43) | 2.11 (1.31, 3.47)** | 1.93 (1.12, 3.39)* |
| Prefer not to say | 3 (75) | 1 (25) | 0.14 (0.01, 1.11) | 0.41 (0.02, 3.63) |
| **Residence** |  |  |  |  |
| Rural | 26 (25) | 78 (75) | Reference | Reference |
| Urban | 79 (24.69) | 241 (75.31) | 1.02 (0.60, 1.68) | 1.51 (0.74, 3.06) |
| **Education** |  |  |  |  |
| Bachelor’s degree or higher | 65 (25.1) | 194 (74.9) | Reference | Reference |
| Lower than bachelor’s degree | 40 (24.24) | 125 (75.76) | 1.05 (0.67, 1.66) | 0.75 (0.36, 1.56) |
| **Occupation** |  |  |  |  |
| Healthcare | 37 (31.62) | 80 (68.38) | Reference | Reference |
| Non-healthcare | 57 (23.85) | 182 (76.15) | 1.48 (0.90, 2.41) | 1.27 (0.67, 2.42) |
| Unemployed | 11 (16.18) | 57 (83.82) | 2.40 (1.16, 5.29)* | 2.11 (0.79, 5.89) |
| **EMR Awareness** |  |  |  |  |
| Have not heard | 17 (18.48) | 75 (81.52) | Reference | Reference |
| Have heard, but not understand | 38 (26.39) | 106 (73.61) | 0.63 (0.33, 1.19) | 0.73 (0.33, 1.57) |
| Understand | 50 (26.6) | 138 (73.4) | 0.63 (0.33, 1.14) | 0.70 (0.28, 1.68) |
| **Health Status** |  |  |  |  |
| Poor | 21 (51.22) | 20 (48.78) | Reference | Reference |
| Well | 84 (21.93) | 299 (78.07) | 3.74 (1.93, 7.26)*** | 2.94 (1.36, 6.38)** |
| **Health Concerns** |  |  |  |  |
| Not concerned | 77 (42.54) | 104 (57.46) | Reference | Reference |
| Concerned | 28 (11.52) | 215 (88.48) | 5.69 (3.51, 9.42)*** | 4.71 (2.80, 8.13)*** |

Table S5. Associations between antecedent factors and PHIPC for improper access.

|  | Low Concerns^a^  (n=95) | High Concerns^a^  (n=329) | Crude OR (95% CI) | Adjusted OR (95% CI) |
| --- | --- | --- | --- | --- |
|  |  |  |  |  |
| **Age Group** |  |  |  |  |
| 18-24 years | 11 (14.86) | 63 (85.14) | Reference | Reference |
| 25-31 years | 41 (21.69) | 148 (78.31) | 0.63 (0.29, 1.27) | 0.61 (0.23, 1.53) |
| 32-38 years | 21 (23.6) | 68 (76.4) | 0.57 (0.25, 1.25) | 0.59 (0.22, 1.57) |
| 39-45 years | 5 (14.29) | 30 (85.71) | 1.05 (0.35, 3.57) | 1.59 (0.45, 6.23) |
| >45 years | 17 (45.95) | 20 (54.05) | 0.21 (0.08, 0.50)*** | 0.35 (0.12, 0.98)* |
| **Sex** |  |  |  |  |
| Female | 63 (25.1) | 188 (74.9) | Reference | Reference |
| Male | 29 (17.16) | 140 (82.84) | 1.62 (1.00, 2.67) | 1.41 (0.82, 2.47) |
| Prefer not to say | 3 (75) | 1 (25) | 0.11 (0.01, 0.89) | 0.24 (0.01, 2.18) |
| **Residence** |  |  |  |  |
| Rural | 22 (21.15) | 82 (78.85) | Reference | Reference |
| Urban | 73 (22.81) | 247 (77.19) | 0.91 (0.52, 1.54) | 1.04 (0.50, 2.11) |
| **Education** |  |  |  |  |
| Bachelor’s degree or higher | 57 (22.01) | 202 (77.99) | Reference | Reference |
| Lower than bachelor’s degree | 38 (23.03) | 127 (76.97) | 0.94 (0.59, 1.51) | 0.73 (0.35, 1.50) |
| **Occupation** |  |  |  |  |
| Healthcare | 31 (26.5) | 86 (73.5) | Reference | Reference |
| Non-healthcare | 53 (22.18) | 186 (77.82) | 1.27 (0.75, 2.10) | 1.29 (0.66, 2.51) |
| Unemployed | 11 (16.18) | 57 (83.82) | 1.87 (0.89, 4.16) | 1.73 (0.64, 4.88) |
| **EMR Awareness** |  |  |  |  |
| Have not heard | 16 (17.39) | 76 (82.61) | Reference | Reference |
| Have heard, but not understand | 40 (27.78) | 104 (72.22) | 0.55 (0.28, 1.03) | 0.64 (0.29, 1.38) |
| Understand | 39 (20.74) | 149 (79.26) | 0.80 (0.41, 1.51) | 1.12 (0.45, 2.77) |
| **Health Status** |  |  |  |  |
| Poor | 19 (46.34) | 22 (53.66) | Reference | Reference |
| Well | 76 (19.84) | 307 (80.16) | 3.49 (1.78, 6.78)*** | 2.44 (1.11, 5.29)* |
| **Health Concerns** |  |  |  |  |
| Not concerned | 70 (38.67) | 111 (61.33) | Reference | Reference |
| Concerned | 25 (10.29) | 218 (89.71) | 5.50 (3.34, 9.31)*** | 4.80 (2.80, 8.46)*** |

Table S6. Associations between antecedent factors and PHIPC for control.

|  | Low Concerns^a^  (n=106) | High Concerns^a^  (n=318) | Crude OR (95% CI) | Adjusted OR (95% CI) |
| --- | --- | --- | --- | --- |
|  |  |  |  |  |
| **Age Group** |  |  |  |  |
| 18-24 years | 17 (22.97) | 57 (77.03) | Reference | Reference |
| 25-31 years | 38 (20.11) | 151 (79.89) | 1.19 (0.61, 2.24) | 1.40 (0.57, 3.37) |
| 32-38 years | 24 (26.97) | 65 (73.03) | 0.81 (0.39, 1.65) | 0.97 (0.38, 2.43) |
| 39-45 years | 8 (22.86) | 27 (77.14) | 1.01 (0.39, 2.73) | 1.41 (0.47, 4.49) |
| >45 years | 19 (51.35) | 18 (48.65) | 0.28 (0.12, 0.65)** | 0.52 (0.19, 1.43) |
| **Sex** |  |  |  |  |
| Female | 70 (27.89) | 181 (72.11) | Reference | Reference |
| Male | 33 (19.53) | 136 (80.47) | 1.59 (1.00, 2.57) | 1.23 (0.72, 2.12) |
| Prefer not to say | 3 (75) | 1 (25) | 0.13 (0.01, 1.03) | 0.36 (0.02, 3.08) |
| **Residence** |  |  |  |  |
| Rural | 25 (24.04) | 79 (75.96) | Reference | Reference |
| Urban | 81 (25.31) | 239 (74.69) | 0.93 (0.55, 1.55) | 1.08 (0.53, 2.16) |
| **Education** |  |  |  |  |
| Bachelor’s degree or higher | 61 (23.55) | 198 (76.45) | Reference | Reference |
| Lower than bachelor’s degree | 45 (27.27) | 120 (72.73) | 0.82 (0.53, 1.29) | 0.89 (0.43, 1.84) |
| **Occupation** |  |  |  |  |
| Healthcare | 36 (30.77) | 81 (69.23) | Reference | Reference |
| Non-healthcare | 55 (23.01) | 184 (76.99) | 1.49 (0.90, 2.43) | 1.80 (0.93, 3.55) |
| Unemployed | 15 (22.06) | 53 (77.94) | 1.57 (0.80, 3.21) | 2.07 (0.79, 5.59) |
| **EMR Awareness** |  |  |  |  |
| Have not heard | 23 (25) | 69 (75) | Reference | Reference |
| Have heard, but not understand | 40 (27.78) | 104 (72.22) | 0.87 (0.47, 1.56) | 1.13 (0.54, 2.36) |
| Understand | 43 (22.87) | 145 (77.13) | 1.12 (0.62, 2.00) | 1.83 (0.76, 4.39) |
| **Health Status** |  |  |  |  |
| Poor | 21 (51.22) | 20 (48.78) | Reference | Reference |
| Well | 85 (22.19) | 298 (77.81) | 3.68 (1.90, 7.15)*** | 2.86 (1.29, 6.36)** |
| **Health Concerns** |  |  |  |  |
| Not concerned | 80 (44.2) | 101 (55.8) | Reference | Reference |
| Concerned | 26 (10.7) | 217 (89.3) | 6.61 (4.05, 11.08)*** | 6.15 (3.61, 10.80)*** |

Table S7. Associations between antecedent factors and PHIPC for awareness.

|  | Low Concerns^a^  (n=101) | High Concerns^a^  (n=323) | Crude OR (95% CI) | Adjusted OR (95% CI) |
| --- | --- | --- | --- | --- |
|  |  |  |  |  |
| **Age Group** |  |  |  |  |
| 18-24 years | 12 (16.22) | 62 (83.78) | Reference | Reference |
| 25-31 years | 44 (23.28) | 145 (76.72) | 0.64 (0.30, 1.26) | 0.59 (0.23, 1.51) |
| 32-38 years | 24 (26.97) | 65 (73.03) | 0.52 (0.23, 1.12) | 0.54 (0.20, 1.46) |
| 39-45 years | 6 (17.14) | 29 (82.86) | 0.94 (0.33, 2.91) | 1.41 (0.41, 5.18) |
| >45 years | 15 (40.54) | 22 (59.46) | 0.28 (0.11, 0.69)** | 0.56 (0.18, 1.69) |
| **Sex** |  |  |  |  |
| Female | 68 (27.09) | 183 (72.91) | Reference | Reference |
| Male | 30 (17.75) | 139 (82.25) | 1.72 (1.07, 2.82)* | 1.44 (0.82, 2.56) |
| Prefer not to say | 3 (75) | 1 (25) | 0.12 (0.01, 0.99) | 0.37 (0.02, 3.53) |
| **Residence** |  |  |  |  |
| Rural | 26 (25) | 78 (75) | Reference | Reference |
| Urban | 75 (23.44) | 245 (76.56) | 1.09 (0.64, 1.80) | 1.33 (0.65, 2.75) |
| **Education** |  |  |  |  |
| Bachelor’s degree or higher | 58 (22.39) | 201 (77.61) | Reference | Reference |
| Lower than bachelor’s degree | 43 (26.06) | 122 (73.94) | 0.82 (0.52, 1.29) | 0.56 (0.27, 1.16) |
| **Occupation** |  |  |  |  |
| Healthcare | 37 (31.62) | 80 (68.38) | Reference | Reference |
| Non-healthcare | 55 (23.01) | 184 (76.99) | 1.55 (0.94, 2.53) | 2.03 (1.03, 4.09)* |
| Unemployed | 9 (13.24) | 59 (86.76) | 3.03 (1.41, 7.13)** | 4.41 (1.54, 13.70)** |
| **EMR Awareness** |  |  |  |  |
| Have not heard | 19 (20.65) | 73 (79.35) | Reference | Reference |
| Have heard, but not understand | 41 (28.47) | 103 (71.53) | 0.65 (0.35, 1.20) | 0.83 (0.38, 1.80) |
| Understand | 41 (21.81) | 147 (78.19) | 0.93 (0.50, 1.70) | 1.65 (0.66, 4.18) |
| **Health Status** |  |  |  |  |
| Poor | 22 (53.66) | 19 (46.34) | Reference | Reference |
| Well | 79 (20.63) | 304 (79.37) | 4.46 (2.30, 8.71)*** | 4.04 (1.82, 9.06)*** |
| **Health Concerns** |  |  |  |  |
| Not concerned | 78 (43.09) | 103 (56.91) | Reference | Reference |
| Concerned | 23 (9.47) | 220 (90.53) | 7.24 (4.37, 12.42)*** | 6.87 (3.93, 12.50)*** |
