# Supplementary material for: Privacy concerns regarding personal health information in Myanmar: A cross-sectional survey in a least developed country
Source: PLOS Digit Health. 2026 Mar 26;5(3):e0001007. doi: 10.1371/journal.pdig.0001007 (PMC13020815; doi:10.1371/journal.pdig.0001007)
Supplement: S1 File — Survey questionnaires in English. (PDF) [file pdig.0001007.s003.pdf]

Personal Health Information Privacy Concerns of Individuals in Myanmar

I am a student of M.Sc. Biomedical and Health Informatics program, Mahidol University, Thailand. The general objective of this survey is to study about personal health information privacy concerns of individuals in Myanmar. Anyone residing in Myanmar, aged 18 or above, and can read and understand Burmese can participate in the survey. The estimated time taken to complete the survey is 10 minutes You can quit at any time during the survey. And your confidentialities will be secured.

For any confusion during the participation of the survey, please contact me via the email hoarfrost.zin@gmail.com or mobile 092057884.

For any inconveniences during the participation of the survey, please contact the Ethics Committee of the Faculty of Tropical Medicine, Mahidol University.

Address: 4th Floor, The 60th Anniversary of His Majesty the King's Accession to the Throne Building, Faculty of Tropical Medicine, Mahidol University, 420/6 Ratchawithi Road, Bangkok 10400, Thailand.

Email: tmectropmed@mahidol.ac.th

Please read below description about personal health information for better comprehension of the questionnaire.

Personal Health Information is all "individually identifiable health information" held or transmitted by a covered entity or its business associate, in any form or media, whether electronic, paper, or oral.

"Individually identifiable health information" is information, including demographic data, that relates to

- the individual's past, present or future physical or mental health or condition,

- the provision of health care to the individual, or

- the past, present, or future payment for the provision of health care to the individual,

and that identifies the individual or for which there is a reasonable basis to believe it can be used to identify the individual. Individually identifiable health information includes many common identifiers (e.g., name, address, birth date, Social Security Number).

* Indicates required question

1. What is your age? *

2. Are you a Myanmar citizen? *

Mark only one oval.

- No
- Yes

3. Do you consent to participate in this survey? *

Mark only one oval.

- No
- Yes

Background Information

4. What is your sex? *

Mark only one oval.

- Male
- Female
- Prefer not to say

5. Where do you live? *

Mark only one oval.

- Urban (living in Wards)
- Rural (living in Village Tracts)

6. What is your highest level of education? *

Mark only one oval.

- No school
- Some school with no degree
- High school graduate
- Some college/university with no degree
- Bachelor's degree
- Postgraduate degree

7. Where do you work at? *

Mark only one oval.

- Healthcare
- Non-healthcare
- Unemployed

8. How do you rate your current health status? *

Mark only one oval.

	1	2	3	4	5	6	7	
Poo								Well

9. How much concerned are you about your health? *

Mark only one oval.

	1	2	3	4	5	6	7	
Not	⬭	⬭	⬭	⬭	⬭	⬭	⬭	Concerned

10. How well do you know about Electronic Medical Records? *

Mark only one oval.

- ⬭ Have not heard
- ⬭ Have heard but not understand
- ⬭ Understand

Personal Health Information Privacy Concerns

Here are some statements about personal health information. From the standpoint of personal privacy please indicate the extent to which you, as an individual, agree or disagree with each statement by selecting the appropriate number. Each of the items is followed by a seven-point Likert scale anchored by “Strongly disagree” (1) and “Strongly agree” (7).

Personal Health Information is all "individually identifiable health information" held or transmitted by a covered entity or its business associate, in any form or media, whether electronic, paper, or oral.

"Individually identifiable health information" is information, including demographic data, that relates to:

- the individual's past, present or future physical or mental health or condition,

- the provision of health care to the individual, or

- the past, present, or future payment for the provision of health care to the individual,
and that identifies the individual or for which there is a reasonable basis to believe it can be used to identify the individual. Individually identifiable health information includes many common identifiers (e.g., name, address, birth date, Social Security Number).

11. It usually bothers me when healthcare entities ask me for personal health information. *

Mark only one oval.

	1	2	3	4	5	6	7	
Stro	◯	◯	◯	◯	◯	◯	◯	Strongly agree

12. When healthcare entities ask me for personal health information, I sometimes think twice before providing it.

Mark only one oval.

	1	2	3	4	5	6	7	
Stro	◯	◯	◯	◯	◯	◯	◯	Strongly agree

13. It bothers me to give personal health information to so many healthcare entities. *

Mark only one oval.

	1	2	3	4	5	6	7	
Stro	◯	◯	◯	◯	◯	◯	◯	Strongly agree

14. I’m concerned that healthcare entities are collecting too much personal health information about me.

Mark only one oval.

	1	2	3	4	5	6	7	
Stro	◯	◯	◯	◯	◯	◯	◯	Strongly agree

15. I am concerned that healthcare entities do not take enough steps to make sure that my personal health information in their files is accurate.

Mark only one oval.

	1	2	3	4	5	6	7	
Stro	◯	◯	◯	◯	◯	◯	◯	Strongly agree

16. I am concerned that healthcare entities do not have adequate procedures to correct errors in my personal health information.

Mark only one oval.

	1	2	3	4	5	6	7	
Stro	◯	◯	◯	◯	◯	◯	◯	Strongly agree

17. I am concerned that healthcare entities do not devote enough time and effort to verifying the accuracy of personal health information in their databases.

Mark only one oval.

	1	2	3	4	5	6	7	
Stro	◯	◯	◯	◯	◯	◯	◯	Strongly agree

18. I'm concerned that when I give personal health information to a healthcare entity for some reason, the entity would use the information for other reasons.

Mark only one oval.

	1	2	3	4	5	6	7	
Stro	◯	◯	◯	◯	◯	◯	◯	Strongly agree

19. I am concerned that healthcare entities would sell my personal health information in their databases to other companies.

Mark only one oval.

	1	2	3	4	5	6	7	
Stro	◯	◯	◯	◯	◯	◯	◯	Strongly agree

20. I am concerned that healthcare entities would share my personal health information with other entities without my authorization.

Mark only one oval.

	1	2	3	4	5	6	7	
Stro	◯	◯	◯	◯	◯	◯	◯	Strongly agree

21. I am concerned that healthcare entities do not devote enough time and effort in preventing unauthorized access to my personal health information.

Mark only one oval.

	1	2	3	4	5	6	7	
Stro	◯	◯	◯	◯	◯	◯	◯	Strongly agree

22. I am concerned that healthcare entities' databases that contain my personal health information are not protected from unauthorized access.

Mark only one oval.

	1	2	3	4	5	6	7	
Stro	◯	◯	◯	◯	◯	◯	◯	Strongly agree

23. I am concerned that healthcare entities do not take enough steps to make sure that unauthorized people cannot access my personal health information in their databases.

Mark only one oval.

	1	2	3	4	5	6	7	
Stro	◯	◯	◯	◯	◯	◯	◯	Strongly agree

24. It usually bothers me when I do not have control or autonomy over decisions about how m personal health information is collected, used and shared by healthcare entities.

Mark only one oval.

	1	2	3	4	5	6	7	
Stro	◯	◯	◯	◯	◯	◯	◯	Strongly agree

25. It usually bothers me when I do not have control of personal health information that I provide to healthcare entities.

Mark only one oval.

	1	2	3	4	5	6	7	
Stro	◯	◯	◯	◯	◯	◯	◯	Strongly agree

26. It usually bothers me when healthcare entities seeking my personal health information do not disclose the way the data were collected, processed, and used.

Mark only one oval.

	1	2	3	4	5	6	7	
Stro	◯	◯	◯	◯	◯	◯	◯	Strongly agree

27. It usually bothers me when I am not aware or knowledgeable about how my personal health information will be used by healthcare entities.

Mark only one oval.

	1	2	3	4	5	6	7	
Stro	◯	◯	◯	◯	◯	◯	◯	Strongly agree

This content is neither created nor endorsed by Google.

Google Forms
