# Supplementary material for: Privacy concerns regarding personal health information in Myanmar: A cross-sectional survey in a least developed country
Source: PLOS Digit Health. 2026 Mar 26;5(3):e0001007. doi: 10.1371/journal.pdig.0001007 (PMC13020815; doi:10.1371/journal.pdig.0001007)
Supplement: S2 File — Survey questionnaires in Myanmar. (PDF) [file pdig.0001007.s004.pdf]

ကျန်းမာရေးနှင့်ဆိုင်သော ကိုယ်ရေးကိုယ်တာ အချက်အလက်များလုံခြုံမှုအတွက် စိုးရိမ်ခြင်း

လေးစားရပါသော မေးခွန်းလွှာဖြေဆိုသူများရှင့်

ဤစစ်တမ်းမှာ ကျွန်မ၏မဟာဘွဲ့ (M.Sc. Biomedical and Health Informatics, Mahidol University, Thailand) အတွက်စာတမ်းပြုစုရန် ကောက်ခံခြင်းဖြစ်ပါသည်။ ဤစာတမ်းပြုစုရခြင်းရည်ရွယ်ချက်မှာ မြန်မာပြည်သူများ၏ မိမိကျန်းမာရေးနှင့်ဆိုင်သော ကိုယ်ရေးကိုယ်တာအချက်အလက်များလုံခြုံရေးအတွက် စိုးရိမ်မှုအဆင့်ကိုတိုင်းတာရန်ဖြစ်ပြီး၊ ထိုမှတဆင့် ၎င်းအချက်အလက်များကို လုံခြုံမှုပေးနိုင်သော ကျန်းမာရေးစောင့်ရှောက်မှုစနစ်များ ပေါ်ပေါက်လာရေးကို အကျိုးပြုရန်ဖြစ်ပါသည်။

အသက် ၁၈ နှစ်နှင့်အထက် မြန်မာနိုင်ငံသူ/သား မည်သူမဆို ပါဝင်ဖြေဆိုနိုင်ပါသည်။

ဖြေဆိုသူ၏ကိုယ်ရေးကိုယ်တာအချက်အလက်များကို သိမ်းဆည်းရယူထားမည်မဟုတ်ပါ။

မရှင်းလင်းသည်များရှိပါက ကျွန်မ၏အီးမေးလ် hoarfrost.zin@gmail.com သို့လည်းကောင်း၊ ဖုန်းနံပါတ် 092057884 သို့လည်းကောင်း ဆက်သွယ်မေးမြန်းနိုင်ပါသည်။

ဤစစ်တမ်းနှင့်ပတ်သက်၍ စိတ်အနှောင့်အယှက်တစုံတရာဖြစ်ခဲ့ရပါက Mahidol တက္ကသိုလ်၊ Tropical Medicine ဌာန၏ Ethics Committee သို့ အောက်ပါလိပ်စာအတိုင်း အကြောင်းကြားနိုင်ပါသည်။

Address: 4th Floor, The 60th Anniversary of His Majesty the King's Accession to the Throne Building, Faculty of Tropical Medicine, Mahidol University, 420/6 Ratchawithi Road, Bangkok 10400, Thailand.

Email: tmectropmed@mahidol.ac.th

ပထမဦးစွာ မေးခွန်းများမဖြေဆိုခင် "ကျန်းမာရေးနှင့်ဆိုင်သော ကိုယ်ရေးကိုယ်တာအချက်အလက်များ" ဆိုသည်ကို နားလည်ထားရန်လိုအပ်သဖြင့် အောက်ပါအနက်ဖွင့်ဆိုချက်ကို ဖတ်ရှုပေးပါရန် မေတ္တာရပ်ခံအပ်ပါသည်။

ကျန်းမာရေးနှင့်ဆိုင်သော ကိုယ်ရေးကိုယ်တာအချက်အလက်များမှာ "မိမိမည်သူမည်ဝါဖြစ်ကြောင်းကိုဖော်ပြနိုင် စွမ်းရှိသော ကျန်းမာရေးဆိုင်ရာအချက်အလက်အားလုံး (all individually identifiable health information)" ကို ဆိုလိုသည်။ ၎င်းအချက်အလက်များသည် electronic နည်းလမ်းဖြင့်ဖြစ်စေ၊ စာရွက်ဖြင့်ဖြစ်စေ၊ နှုတ်စကားဖြင့် ဖြစ်စေ သိမ်းဆည်းသော/ဖြန့်ဝေသောအချက်အလက်များအားလုံး အကျုံးဝင်သည်။

ထိုအချက်အလက်များထဲတွင် အသက်၊ ကျား/မ စသည့် demographic အချက်အလက်များအပြင် အောက်ပါတို့လည်းပါဝင်သည်။

- မိမိ၏ အတိတ်၊ ပစ္စုပ္ပန်၊ အနာဂါတ်ကာလရှိ ကိုယ်/စိတ်ကျန်းမာရေးအခြေအနေဆိုင်ရာအချက်အလက်များ

- မိမိခံယူခဲ့သော/ခံယူလျက်ရှိသော/ခံယူမည့် ကျန်းမာရေးစောင့်ရှောက်မှုနှင့်ဆိုင်သောအချက်အလက်များ

- ထိုကျန်းမာရေးစောင့်ရှောက်မှုများအတွက် ငွေပေးချေခြင်းဆိုင်ရာအချက်အလက်များ

- ပုံမှန်ပေးနေကျဖြစ်သော အမည်၊ မွေးနေ့၊ လိပ်စာ၊ မှတ်ပုံတင်နံပါတ် စသည့်မိမိဖြစ်သူမည်ဝါဖြစ်ကြောင်း (identity) ကိုဖော်ပြနိုင်သော အချက်အလက်အားလုံးကို ဆိုလိုခြင်းဖြစ်သည်။

* Indicates required question

1. အသက် (အင်္ဂလိပ်နံပါတ်ဖြင့်ဖော်ပြပါ - ဥပမာ 25) *

2. မြန်မာနိုင်ငံသားဟုတ်ပါသလား
(အဖြေတခုသာ အမှန်ခြစ်ပါ)

- [ ] မဟုတ်ပါ
- [ ] ဟုတ်ပါသည်

3. ဤစစ်တမ်းတွင်ပါဝင်ဖြေဆိုရင် သဘောတူပါသလား *
(အဖြေတခုသာ အမှန်ခြစ်ပါ)

- [ ] သဘောမတူပါ (သဘောမတူလျှင် ဆက်လက်မဖြေဆိုဘဲရပ်တန့်နိုင်ပါသည်)
- [ ] သဘောတူပါသည်

Background Information

4. ကျား/မ *
(အဖြေတခုသာ အမှန်ခြစ်ပါ)

- [ ] ကျား
- [ ] မ
- [ ] မဖော်ပြလိုပါ

5. နေထိုင်ရာဒေသ *
(အဖြေတခုသာ အမှန်ခြစ်ပါ)

- မြို့ပြ (ရပ်ကွက်တွင်နေထိုင်သူ)
- ကျေးလက် (ကျေးရွာတွင်နေထိုင်သူ)

6. အမြင့်ဆုံးပြီးမြောက်ခဲ့သောပညာရေး *
(အဖြေတခုသာ အမှန်ခြစ်ပါ)

- ကျောင်းမနေဖူးပါ
- ကျောင်းနေဖူးသည်၊ အထက်တန်းမအောင်သေးပါ
- အထက်တန်းအောင်
- ကောလိပ်/တက္ကသိုလ်တက်ဖူးသည်၊ ဘွဲ့မရသေးပါ
- Bachelor ဘွဲ့ရ
- မဟာဘွဲ့/PhD ဘွဲ့ရ

7. အလုပ်အကိုင် *
(အဖြေတခုသာ အမှန်ခြစ်ပါ)

- မရှိပါ
- ကျန်းမာရေးစောင့်ရှောက်မှုနှင့်ဆိုင်သောအလုပ်
- အခြားအလုပ်

8. မိမိ၏လက်ရှိကျန်းမာရေးအခြေအနေကို အဆင့်သတ်မှတ်ပြပါ *
(နံပါတ်တခုသာ အမှန်ခြစ်ပါ)

	1	2	3	4	5	6	7	
မကောင်းပါ								ကောင်းသည်

9. မိမိလက်ရှိကျန်းမာရေးအတွက် မည်မျှစိုးရိမ်ပူပန်သည်ကို အဆင့်သတ်မှတ်ပြပါ *
(နံပါတ်တခုသာ အမှန်ခြစ်ပါ)

	1	2	3	4	5	6	7	
မပူပန်ပါ	◯	◯	◯	◯	◯	◯	◯	ပူပန်သည်

10. သင်အီလက်ထရောနစ်ကျန်းမာရေးမှတ်တမ်းများ (electronic medical records) အကြောင်း မည်မျှ သိသလဲ
(အဖြေတခုသာ အမှန်ခြစ်ပါ)

- ◯ မကြားဖူးပါ
- ◯ ကြားဖူးသည်၊ နားမလည်ပါ
- ◯ နားလည်ပါသည်

11. ကျန်းမာရေးစောင့်ရှောက်မှုဌာနများ (ဆေးရုံ/ဆေးခန်းစသည်) မှ မိမိ၏ကိုယ်ရေးကိုယ်တာ ကျန်းမာရေးအချက်အလက်များမေးမြန်းခြင်းသည် များသောအားဖြင့် မိမိအား စိတ်အနှောင့်အယှက်ဖြစ်စေပါသည်။
(နံပါတ်တခုသာ အမှန်ခြစ်ပါ)

	1	2	3	4	5	6	7	
လုံးဝသဘောမတူပါ	◯	◯	◯	◯	◯	◯	◯	လုံးဝသဘောတူသည်

12. ကျန်းမာရေးစောင့်ရှောက်မှုဌာနများမှ မိမိ၏ကိုယ်ရေးကိုယ်တာကျန်းမာရေးအချက်အလက်များ မေးမြန်းလျှင် တခါတရံတွင် မဖြေဆိုမီ ၂ခါပြန်စဉ်းစားလေ့ရှိပါသည်။
(နံပါတ်တခုသာ အမှန်ခြစ်ပါ)

	1	2	3	4	5	6	7	
လုံးဝသဘောမတူပါ	◯	◯	◯	◯	◯	◯	◯	လုံးဝသဘောတူသည်

13. ကျန်းမာရေးစောင့်ရှောက်မှုဌာနအများအပြားသို့ မိမိ၏ကိုယ်ရေးကိုယ်တာကျန်းမာရေး အချက်အလက်များပေးရခြင်းသည် မိမိအား စိတ်အနှောင့်အယှက်ဖြစ်စေပါသည်။
(နံပါတ်တခုသာ အမှန်ခြစ်ပါ)

	1	2	3	4	5	6	7	
လုံးဝသဘောမတူပါ	◯	◯	◯	◯	◯	◯	◯	လုံးဝသဘောတူသည်

14. ကျန်းမာရေးစောင့်ရှောက်မှုဌာနများသည် မိမိ၏ကိုယ်ရေးကိုယ်တာကျန်းမာရေးအချက်အလက် များကို အလွန်အကျွံကောက်ယူနေသည်ဟု စိုးရိမ်မိပါသည်။
(နံပါတ်တခုသာ အမှန်ခြစ်ပါ)

	1	2	3	4	5	6	7	
လုံးဝသဘောမတူပါ	◯	◯	◯	◯	◯	◯	◯	လုံးဝသဘောတူသည်

15. ကျန်းမာရေးစောင့်ရှောက်မှုဌာနများသည် ၎င်းတို့၏ဖိုင်များအတွင်းရှိ မိမိ၏ကိုယ်ရေးကိုယ်တာကျန်းမာရေးအချက်အလက်များအား မှန်ကန်တိကျစေရန်လုပ်ဆောင်သင့်သည်တို့ကို လုံလောက်စွာလိုက်နာခြင်းမရှိဟု မိမိအနေဖြင့်စိုးရိမ်မိပါသည်။
(နံပါတ်တခုသာ အမှန်ခြစ်ပါ)

	1	2	3	4	5	6	7	
လုံးဝသဘောမတူပါ	◯	◯	◯	◯	◯	◯	◯	လုံးဝသဘောတူသည်

16. ကျန်းမာရေးစောင့်ရှောက်မှုဌာနများတွင် မိမိ၏ကိုယ်ရေးကိုယ်တာကျန်းမာရေးအချက်အလက်များ အမှားအယွင်းရှိခဲ့လျှင် ပြန်လည်ပြင်ဆင်ရန် လုံလောက်သောလုပ်ထုံးလုပ်နည်းများမရှိဟု စိုးရိမ်မိပါသည်။
(နံပါတ်တခုသာ အမှန်ခြစ်ပါ)

	1	2	3	4	5	6	7	
လုံးဝသဘောမတူပါ	◯	◯	◯	◯	◯	◯	◯	လုံးဝသဘောတူသည်

17. ကျန်းမာရေးစောင့်ရှောက်မှုဌာနများသည် ၎င်းတို့၏အချက်အလက်သိုလှောင်ရာနေရာ (database) များတွင်သိမ်းဆည်းထားသော မိမိ၏ကိုယ်ရေးကိုယ်တာကျန်းမာရေးအချက်အလက်များ မှန်ကန်မှုရှိမရှိအတည်ပြုရန် လုံလောက်သောအချိန်နှင့် အားစိုက်ထုတ်မှုမရှိဟု မိမိအနေဖြင့်စိုးရိမ်မိပါသည်။
(နံပါတ်တခုသာ အမှန်ခြစ်ပါ)

	1	2	3	4	5	6	7	
လုံးဝသဘောမတူပါ	◯	◯	◯	◯	◯	◯	◯	လုံးဝသဘောတူသည်

18. အကြောင်းတခုခုအတွက် ကျန်းမာရေးစောင့်ရှောက်မှုဌာနတစ်ခုတွင်ပေးအပ်ထားသော မိမိ၏ကိုယ်ရေးကိုယ်တာကျန်းမာရေးအချက်အလက်များကို ၎င်းဌာနမှ အခြားရည်ရွယ်ချက်များ အတွက်အသုံးပြုမည်ကို စိုးရိမ်မိပါသည်။
(နံပါတ်တခုသာ အမှန်ခြစ်ပါ)

	1	2	3	4	5	6	7	
လုံးဝသဘောမတူပါ	◯	◯	◯	◯	◯	◯	◯	လုံးဝသဘောတူသည်

19. ကျန်းမာရေးစောင့်ရှောက်မှုဌာနများသည် ၎င်းတို့သိမ်းဆည်းထားသော မိမိ၏ကိုယ်ရေးကိုယ်တာ ကျန်းမာရေးအချက်အလက်များအား အခြားဌာနများသို့ရောင်းချမည်ကို စိုးရိမ်မိပါသည်။
(နံပါတ်တခုသာ အမှန်ခြစ်ပါ)

	1	2	3	4	5	6	7	
လုံးဝသဘောမတူပါ	◯	◯	◯	◯	◯	◯	◯	လုံးဝသဘောတူသည်

20. ကျန်းမာရေးစောင့်ရှောက်မှုဌာနများသည် မိမိ၏ကိုယ်ရေးကိုယ်တာကျန်းမာရေးအချက်အလက် များကို မိမိ၏ခွင့်ပြုချက်မပါရှိဘဲ အခြားဌာနများနှင့်မျှဝေမည်ကို စိုးရိမ်မိပါသည်။
(နံပါတ်တခုသာ အမှန်ခြစ်ပါ)

	1	2	3	4	5	6	7	
လုံးဝသဘောမတူပါ	◯	◯	◯	◯	◯	◯	◯	လုံးဝသဘောတူသည်

21. ကျန်းမာရေးစောင့်ရှောက်မှုဌာနများသည် မိမိ၏ကိုယ်ရေးကိုယ်တာကျန်းမာရေးအချက်အလက်များအား ခွင့်ပြုချက်မရှိဘဲတပါးသူရယူခြင်းမှတားဆီးရန် လုံလောက်သောအချိန်နှင့် အားစိုက်ထုတ်မှုမရှိဟု စိုးရိမ်မိပါသည်။

(နံပါတ်တခုသာ အမှန်ခြစ်ပါ)

	1	2	3	4	5	6	7	
လုံးဝသဘောမတူပါ	◯	◯	◯	◯	◯	◯	◯	လုံးဝသဘောတူသည်

22. ကျန်းမာရေးစောင့်ရှောက်မှုဌာနများရှိ မိမိ၏ကိုယ်ရေးကိုယ်တာကျန်းမာရေးအချက်အလက်များသိမ်းဆည်းထားသော database များကို ခွင့်ပြုချက်မရှိဘဲတပါးသူရယူခြင်းမှ ကာကွယ်ထားခြင်း[illegible] ရှိဟု စိုးရိမ်မိပါသည်။

(နံပါတ်တခုသာ အမှန်ခြစ်ပါ)

	1	2	3	4	5	6	7	
လုံးဝသဘောမတူပါ	◯	◯	◯	◯	◯	◯	◯	လုံးဝသဘောတူသည်

23. ကျန်းမာရေးစောင့်ရှောက်မှုဌာနများသည် ၎င်းတို့၏အချက်အလက်သိုလှောင်ရာနေရာ (database) များတွင်သိမ်းဆည်းထားသော မိမိ၏ကိုယ်ရေးကိုယ်တာကျန်းမာရေးအချက်အလက်များကို ခွင့်ပြုချက်မရှိသူများရယူခြင်းမရှိစေရန် လုံလောက်သောအဆင့်များလုပ်ဆောင်ခြင်းမရှိပ[illegible] စိုးရိမ်မိပါသည်။

(နံပါတ်တခုသာ အမှန်ခြစ်ပါ)

	1	2	3	4	5	6	7	
လုံးဝသဘောမတူပါ	◯	◯	◯	◯	◯	◯	◯	လုံးဝသဘောတူသည်

24. ကျန်းမာရေးစောင့်ရှောက်မှုဌာနများမှ မိမိ၏ကိုယ်ရေးကိုယ်တာကျန်းမာရေးအချက်အလက်များကို ကောက်ယူခြင်း၊ အသုံးပြုခြင်းနှင့် မျှဝေခြင်းများကို မိမိ၏ထိန်းချုပ်မှုမပါဝင်ဘဲ ပြုလုပ်ခြင်းများသည် များသောအားဖြင့် မိမိအား စိတ်အနှောင့်အယှက်ဖြစ်စေပါသည်။

(နံပါတ်တခုသာ အမှန်ခြစ်ပါ)

	1	2	3	4	5	6	7	
လုံးဝသဘောမတူပါ	◯	◯	◯	◯	◯	◯	◯	လုံးဝသဘောတူသည်

25. ကျန်းမာရေးစောင့်ရှောက်မှုဌာနများသို့ပေးထားသော မိမိ၏ကိုယ်ရေးကိုယ်တာကျန်းမာရေး အချက်အလက်များကို ကိုယ်တိုင်ထိန်းချုပ်ပိုင်ခွင့်မရှိခြင်းသည် မိမိအား စိတ်အနှောင့်အယှက်ဖြစ်စေပါသည်။

(နံပါတ်တခုသာ အမှန်ခြစ်ပါ)

	1	2	3	4	5	6	7	
လုံးဝသဘောမတူပါ	◯	◯	◯	◯	◯	◯	◯	လုံးဝသဘောတူသည်

26. ကျန်းမာရေးစောင့်ရှောက်မှုဌာနများသည် မိမိ၏ကိုယ်ရေးကိုယ်တာကျန်းမာရေးအချက်အလက်များကို မည်သို့စုဆောင်း၊ စီမံ၊ အသုံးပြုသည့်အကြောင်း ထုတ်ဖော်မရှင်းလင်းသည့်အခါ များသော အားဖြင့် မိမိအား စိတ်အနှောင့်အယှက်ဖြစ်စေပါသည်။

(နံပါတ်တခုသာ အမှန်ခြစ်ပါ)

	1	2	3	4	5	6	7	
လုံးဝသဘောမတူပါ	◯	◯	◯	◯	◯	◯	◯	လုံးဝသဘောတူသည်

27. မိမိ၏ကိုယ်ရေးကိုယ်တာကျန်းမာရေးအချက်အလက်များကို ကျန်းမာရေးစောင့်ရှောက်မှုဌာနများမှ မည်သို့အသုံးပြုမည်ကို မိမိနားလည်သဘောမပေါက်သောအခါ များသောအားဖြင့် စိတ်အနှောင့်အယှက်ဖြစ်ရပါသည်။

(နံပါတ်တခုသာ အမှန်ခြစ်ပါ)

	1	2	3	4	5	6	7	
လုံးဝသဘောမတူပါ	◯	◯	◯	◯	◯	◯	◯	လုံးဝသဘောတူသည်

This content is neither created nor endorsed by Google.

Google Forms
