# Supplementary material for: Privacy concerns regarding personal health information in Myanmar: A cross-sectional survey in a least developed country
Source: PLOS Digit Health. 2026 Mar 26;5(3):e0001007. doi: 10.1371/journal.pdig.0001007 (PMC13020815; doi:10.1371/journal.pdig.0001007)
Supplement: Attachment — Submitted filename: Response to Reviewers.pdf [file pdig.0001007.s007.pdf]

Responses to Reviewer Comments

Reviewer #1: This study provides meaningful and original insight into digital health privacy concerns. Its findings have practical implications for policymakers, developers, and researchers interested in digital trust and ethical health data use.

To further strengthen the manuscript, I suggest the authors:

Emphasize the sampling limitations (urban and educated bias) more prominently in the Abstract and Limitations section.

Response: Thank you for this helpful suggestion. We agree that the sampling limitations should be stated more prominently. In the abstract, we added a sentence explicitly noting that recruitment via online channels and private clinics may over-represent urban and more educated participants, limiting generalizability (Abstract, Page 2, Lines 44-45). We also added a subheading "Limitations", where we revised it to more clearly highlight the potential urban/educated bias introduced by convenience sampling and to clarify that findings may not generalize to rural or lower-literacy populations (Limitations, Page 34, Lines 592-597).

Clarify the translation validation process (like how conceptual equivalence was verified beyond back-translation).

Response: Thank you for this important comment. We agree that conceptual equivalence is a key concern when adapting privacy-related constructs across languages and health-system contexts. In our study, we used a structured forward–backward translation approach guided by published cross-cultural adaptation recommendations. Beyond back-translation alone, conceptual equivalence was supported through several steps embedded in our process: (1) forward translators were bilingual and familiar with Myanmar's healthcare context, which helped ensure that wording choices reflected locally meaningful expressions of the intended constructs; (2) the two independent forward translations were synthesized through reviewer comparison, allowing ambiguous or overly literal phrasing to be identified and corrected; (3) the back-translation was performed by independent translators blinded to the original English instrument, and the reconciled back-translation was systematically compared with the original to identify not only semantic discrepancies but also potential cultural mismatches or conceptual inconsistencies, which were then resolved through consensus discussion by bilingual reviewers with Myanmar healthcare experience; and (4) we conducted pilot testing in which participants provided feedback on comprehension and ease of understanding, and we made minor wording adjustments to improve clarity while retaining intended meaning. Taken together, these steps provided multiple checkpoints to preserve item intent and reduce the risk of meaning drift during adaptation.

Provide exact details on the data files shared in the supplementary material.

Response: Thank you for this comment. We have expanded the Supporting Information to provide exact details of the shared data file, including file name, format, worksheet structure, sample size, and the variables/items contained in each sheet (Supporting information, Page 39, Lines 704-708).

Consider a few language refinements for readability.

Response: Thank you for this suggestion. We have carefully reviewed the manuscript and made minor language refinements throughout. These edits do not alter the scientific content but enhance the flow and readability.

Reviewer #2: Overall Assessment: The manuscript addresses a timely and understudied topic. Privacy concerns around personal health information are critical in least developed countries like Myanmar, especially during the shift to digital health systems. The study fills an important gap. It uses a validated scale and provides clear findings on high concern levels and associated factors. The results are relevant for policy in Myanmar and similar settings. However, the sampling method limits representativeness. Reporting of methods and results needs more detail. The work is solid but requires revisions for clarity and rigor.

Strengths: The topic is highly relevant. Few studies exist on health data privacy in least developed countries. The use of a validated 21-item scale with six domains is appropriate. Confirmatory factor analysis adds credibility. The high response rate (77.83% with high concerns) and clear domain differences are interesting. Associations with age, self-reported health, and health worries are meaningful. Implications for trust-building and legal frameworks are practical.

Major Concerns:

1) The sample uses convenience methods. Recruitment happened mainly via Facebook and two private clinics. Participants are mostly urban (75%), female (59%), and well-educated (61% bachelor's degree or higher). Many work in healthcare (28%). Younger adults are over-represented. This group likely has higher digital literacy and privacy awareness. Rural, older, less-educated, and low-income people are under-represented. Findings cannot speak for all of Myanmar. The authors must stress this limitation and present results as exploratory for accessible populations.

Response: Thank you for this careful and detailed comment. We agree that the convenience sampling approach and recruitment strategy require prominent emphasis, and that the findings should be framed as exploratory for more accessible populations. Accordingly, in the Abstract we added a sentence explicitly noting that recruitment via online channels and private clinics may over-represent urban, more educated, and digitally connected participants, limiting generalizability (Abstract, Page 2, Lines 44-45). We also created a separate "Limitations" subheading and revised this section to more clearly describe the resulting urban/educated bias and to state explicitly that the reported prevalence estimates and associations should be interpreted as exploratory/hypothesis-generating, reflecting privacy concerns among digitally connected and clinic-accessing groups rather than the Myanmar population as a whole (Limitations, Page 34, Lines 592-597).

2) Political instability after the 2021 coup is mentioned briefly. Internet restrictions and safety fears probably affected who could join online. No major incidents occurred during data collection, but the context still matters. Expand discussion on how the situation may influence responses and access.

Response: Thank you for highlighting the importance of the broader political and security environment. We have expanded the Limitations to discuss how post-coup internet disruptions/restrictions and safety concerns may have affected access to the online survey (and thus participation) and may also have influenced privacy attitudes and responses, while noting that these factors were not directly measured (Limitations, Page 35-36, Lines 628-634).

3) Regression results show wide confidence intervals in some subgroups. This reflects small subgroup sizes. Interpretation should stay cautious.

Response: Thank you for noting this. We agree that some stratified estimates in Table 5 have wide confidence intervals, indicating limited precision. As we had already acknowledged in the Limitations that the sample size was not calculated to support subgroup analyses and that statistical power for stratified models may be limited, we have now also revised the Results text to explicitly flag the specific subgroup estimates with wide confidence intervals as imprecise (Results, Page 23, Lines 456-457).

4) Details on recruitment are missing. How many were invited online versus in clinics? These points affect reproducibility and bias assessment.

Response: Thank you for this important point. We agree that reporting recruitment flow by channel would strengthen reproducibility and assessment of selection bias. In our study, recruitment occurred through social media dissemination and two private clinics; however, because all respondents completed the same Google Form and recruitment source was not recorded, we are unable to quantify how many participants were invited or enrolled via online versus clinic-based recruitment. We have added a limitation noting that the absence of channel tracking prevents disaggregation of response counts and limits channel-specific bias assessment (Limitations, Page 35, Lines 621-623).

5) The manuscript uses only tables for results. This works but feels dense. Other visualizations would make patterns easier to grasp.

Response: Thank you for this valuable suggestion. We agree that visual representation can enhance clarity and improve readers' ability to interpret regression findings. In response, we have added a new forest plot (Figure 3, Page 30) to visually summarize the crude and adjusted odds ratios with corresponding 95% confidence intervals for factors associated with high PHIPC. This figure complements Table 5 and Supplementary Table S1 by illustrating the direction, magnitude, and precision of associations on a logarithmic scale. The figure interpretation has been inserted in the Results section immediately following the description of the multivariable logistic regression results (Results, Page 22, Lines 447-449). We believe this addition improves readability while retaining detailed tabular information for reference.

Recommendations:

Can you compare the statistics among social media vs clinic statistics? The scenario could be different and can be good addition to the paper. Acknowledge sampling limitations explicitly. Frame the study as exploratory among accessible urban and clinic-based adults. Add recruitment details (numbers approached, response rates by method). Discuss the political and digital access context in limitations. Strengthen the discussion of implications for rural and marginalized groups.

Response: Thank you for these integrated recommendations. The issues raised have been addressed in our revisions as detailed in our point-by-point responses to comments 1-5 above. Regarding the suggestion to compare participants recruited via social media versus clinics and to report channel-specific response rates, recruitment source was not captured at the individual level because all participants completed the same Google Form and recruitment channel was not recorded. Therefore, we were unable to disaggregate statistics or calculate response rates by recruitment method. We have explicitly acknowledged this as a limitation in the revised manuscript.

Reviewer #3: This manuscript addresses an important and timely topic in digital health by examining personal health information privacy concerns in a low-resource (LDC). Overall, the manuscript is clear and informative, with well-presented results supported by organized tables and figures. The discussion effectively places the findings within existing literature, and the conclusions are appropriately drawn from the results. The authors also demonstrate transparency by clearly reporting study limitations and providing practical recommendations for stakeholders. The effort to align with open science principles by indicating how underlying data and tables can be accessed is appreciated.

The comments below are mainly minor and focus on improving clarity, consistency, and organization.

Greater alignment between the abstract and the Objectives section would strengthen the manuscript. The main objective stated in the abstract should be clearly reflected as the primary objective throughout the paper, with consistent use of terminology (e.g., using either "extent" or "level" of privacy concerns consistently). If both primary and secondary objectives are intended, these should be clearly separated and simply stated.

Response: Thank you for this helpful suggestion. We revised the Objectives section to align with the abstract and to present the study aims more clearly. Specifically, we now state two primary objectives explicitly and concisely (Objectives, Page 9, Lines 200-202). We also standardized terminology throughout the manuscript by consistently using the term "level" (rather than alternating between "extent" and "level") to improve clarity and consistency across the abstract and main text.

In the Methods section, minor reorganization would improve readability. The Study Design subsection could briefly explain why a cross-sectional online survey is appropriate for achieving the study aim, rather than restating the objective. Separating the setting, data collection, recruitment, and sampling strategy into distinct subsections would also enhance clarity. In addition, details related to instrument

validation (such as confirmatory factor analysis) would be more appropriately placed in the Data Collection Tool/Instruments section rather than in the Data Analysis section.

Response: Thank you for this constructive and helpful suggestion. We have reorganized the Methods section to improve clarity, readability, and structural coherence. Specifically, The Methods section has been restructured into clearly defined subsections, including study design, setting, participants, study size, assessments, translation process, instrument validation, data analysis, and ethical considerations, to enhance readability and logical flow (Methods, Page 9-15). The study design subsection has been revised to briefly justify the appropriateness of a cross-sectional online survey (Study Design, Page 9-10, Lines 207-222). Details related to instrument validation, including confirmatory factor analysis and reliability assessment, have been relocated from the data analysis section to a dedicated Instrument Validation subsection, as recommended (Instrument Validation, Page 13, Lines 311-320). We believe these revisions improve the organization and transparency of the methodological description while maintaining consistency with reporting standards.

A few additional clarifications would further improve accessibility. Naming the private clinics involved in participant recruitment, instead of referring only to cities, would improve transparency. Defining abbreviations at first mention or moving the abbreviations list earlier in the manuscript would also enhance readability.

Response: Thank you for this thoughtful suggestion. While we agree that specifying clinic names could enhance transparency, we have chosen not to disclose the names of the private outpatient clinics due to ethical and contextual considerations. The consent obtained from the clinics did not explicitly state that institutional names would be publicly reported. That is why we have retained the description of clinic type (private outpatient clinics) and location (township names) without identifying them by name. All abbreviations (e.g., PHI, PHIPC, EMR, CFA) are defined at first mention in the manuscript to enhance readability. In accordance with journal formatting requirements, the consolidated abbreviations list is placed at the end of the manuscript.

Finally, the manuscript would benefit from a brief section explicitly highlighting the study's key contributions to the field.

Response: Thank you for this helpful recommendation. To improve clarity and highlight the study's contribution, we have separated the final paragraph of the Discussion into a distinct subsection titled "Implications." This section explicitly outlines the study's contributions to digital health research in low-resource settings, including its role in establishing baseline evidence on PHIPC in Myanmar and informing privacy governance and digital health policy (Implications, Page 33, Lines 574-585).

Overall, these are minor, addressable points. Addressing them would further strengthen the clarity and organization of an already solid and valuable contribution to digital health research.

FEEDBACK

Response: Thank you for summarizing. They are addressed in our detailed responses above. For ease of reference, we also provide brief consolidated responses below.

1. Abstract

The abstract provides a clear and concise summary of the manuscript, effectively presenting the research problem, study objective, methodology, key findings, and conclusions within a limited word count. The authors have adhered to the journal's abstract length requirement, with a total of 282 words, which is appropriate. Importantly, the abstract is well aligned with the content of the full manuscript, accurately reflecting the study's scope and main contributions.

2. Background and introduction

I appreciate the clarity and accessibility with which the problem is presented in the Background and Introduction. The rationale for why this topic matters is clearly articulated, allowing readers to easily grasp the importance of personal health information (PHI) privacy. The writing is coherent, concise, and logically structured, with a smooth flow of ideas and effective comparisons drawn from related research conducted in Europe (Ireland), Oceania (New Zealand), Asia, Africa, and the United States. These comparisons help illustrate the magnitude of privacy concerns surrounding personal health information (PHI) and demonstrate how the topic adds knowledge and contributes meaningfully to digital health research.

In addition, the authors use clear and non-technical language, avoiding unnecessary jargon, which makes the manuscript accessible not only to medical professionals but also to a broader readership. This is a notable strength of the paper. Furthermore, each key idea is appropriately supported by references, demonstrating a thorough engagement with relevant literature and strengthening the credibility and scholarly rigor of the manuscript.

3. Study Objectives

While the Objectives section identifies the primary goal of the study, there is an inconsistency in the terminology used to describe it. The manuscript alternates between terms such as "extent" and "level" of personal health information privacy concerns (PHIPC). To avoid ambiguity, the authors should select one term and use it consistently across the abstract, objectives, and throughout the manuscript.

Response: Thank you for this helpful suggestion. We have now standardized terminology throughout the manuscript by consistently using the term "level" (rather than alternating between "extent" and "level") to improve clarity and consistency across the abstract and main text.

In addition, the Objectives section includes extensive narrative explanations and contextual framing (e.g., references to differences between developed and developing countries and political instability). While relevant, these explanations reduce the clarity of the primary objective. Such contextual information would be more appropriately placed in the Background or Discussion sections.

Response: Thank you for this helpful suggestion. We have revised the Objectives section to remove contextual narrative and present the study aims concisely and directly (Objectives, Page 9, Lines 200-202). We believe this revision improves clarity, focus, and structural coherence.

To strengthen coherence, the authors are encouraged to present the main objective concisely and in direct alignment with the abstract, retaining the action verb "assess not this study aimed to investigate PHIPC…." If both primary and secondary objectives are intended, these should be clearly separated and simply stated, with the objective described in the abstract presented as the primary objective and any additional aims framed as secondary.

Response: Thank you for this helpful suggestion. We revised the Objectives section to align with the abstract and to present the study aims more clearly. Specifically, we now state two primary objectives explicitly and concisely (Objectives, Page 9, Lines 200-202).

4. Methodology:

a. Study design: The cross-sectional study design is appropriate for the study objectives. However, in the Study Design section, the authors restate the study objective (i.e., assessing the extent of PHI privacy concerns and identifying associated factors) rather than briefly explaining how the study design is suitable for achieving this aim. The authors may consider concisely justifying the choice of a cross-sectional design (e.g., its suitability for measuring the prevalence and associated factors of PHIPC at a single point in time) instead of reiterating the objective.

Response: Thank you for this constructive and helpful suggestion. The study design subsection has been revised to briefly justify the appropriateness of a cross-sectional online survey instead of rewriting the objectives (Study Design, Page 9-10, Lines 207-222).

Please consider naming the private clinics where participants with low digital literacy were recruited, rather than referring only to the cities, to improve clarity.

Response: Thank you for this thoughtful suggestion. While we agree that specifying clinic names could enhance transparency, we have chosen not to disclose the names of the private outpatient clinics due to ethical and contextual considerations. The consent obtained from the clinics did not explicitly state that institutional names would be publicly reported. That is why we have retained the description of clinic type (private outpatient clinics) and location (township names) without identifying them by name.

b. Setting: In the Setting section, information related to the study setting, data collection process, and participant recruitment is combined. For improved clarity and structure, the authors may consider separating these elements into distinct subsections (e.g., Setting, Data Collection, and Recruitment).

Response: Thank you for this helpful suggestion. We carefully reviewed the organization of the Methods section in response to this comment. The Setting subsection currently describes the geographic and contextual background of the study, while participant eligibility and sampling are presented separately under the Participants subsection. Because recruitment procedures are closely tied to the study context and platforms used, we elected to retain this information within the Setting subsection to preserve narrative coherence and avoid unnecessary fragmentation of closely related content. We believe the current structure maintains clarity while ensuring logical flow across subsections.

c. Participants: The use of convenience sampling is understandable given the political instability in the country and the reliance on online data collection to facilitate participation. However, to improve clarity, the authors are encouraged to separate the descriptions of participants' eligibility criteria, recruitment process, and sampling strategy into distinct subsections.

Response: Thank you for this helpful suggestion. As noted in our response to Comment (b) above, we carefully reviewed the structure of the Methods section to ensure clarity and logical separation of methodological components. Eligibility criteria are presented under the Participants subsection, recruitment procedures are described within the Setting subsection, and the convenience sampling strategy is stated under Participants. We believe this organization sufficiently distinguishes eligibility, recruitment, and sampling while preserving narrative coherence. Therefore, we have retained the current structure.

d. Data Collection Tool/Instruments: The assessment section provides a clear and detailed description of the data collection tools. However, the description of tool validation using confirmatory factor analysis (CFA) is currently placed under the data analysis section. The authors are encouraged to move this description to the Data Collection Tool/Instruments section, as it more appropriately reflects instrument validation.

Response: Thank you for this constructive and helpful suggestion. Details related to instrument validation, including confirmatory factor analysis and reliability assessment, have been relocated from the data analysis section to a dedicated Instrument Validation subsection, as recommended (Instrument Validation, Page 13, Lines 311-320).

e. Translation process/data collection process: procedures are clearly and well described. The manuscript provides a transparent explanation of how information was translated between the local language and English, and the data validation and quality control measures appear to be robust.

f. Data Analysis: The data analysis section is well described and easy to follow. However, the statement describing the validation of the questionnaire using confirmatory factor analysis (CFA) is not essential in this section and would be more appropriately placed under the Data Collection Tool/Instruments section.

NB: Responses from Google Forms were exported as CSV files and analyzed using RStudio (version 2024.12.0+467). Could be a good introductory sentence in data analysis.

Response: Thank you for this helpful suggestion. We have revised the first sentence of the Data Analysis subsection to clearly state the data export format (XLSX file) and software used for analysis (Data Analysis, Page 13, Line 323). The XLSX format was selected to ensure accurate retention of Burmese characters during data processing. We believe this clarification enhances methodological transparency and reproducibility.

g. Ethical Approval: This section was well stated, and this paper fulfilled ethical requirements and protected participants' privacy and protection.

5. Results, Discussions, Evidence, and Conclusions:

The Results section is clearly presented, with well-organized tables and figures that appropriately address the study objectives. The findings are logically arranged and easy to follow.

The Discussion section is coherent and thoughtfully written, with effective comparisons to related studies. These comparisons clearly highlight the significance of the findings, as well as similarities and differences with previous research.

I also appreciate the transparent and comprehensive reporting of study limitations. The authors clearly acknowledge key limitations related to the study design, sample size, subgroup imbalances resulting from online data collection, and the underrepresentation of rural populations (given that approximately 70% of the country is rural, while only about 25% of participants were included). This level of transparency is valuable for readers and future researchers in identifying gaps and areas for improvement.

As a suggestion, the manuscript would benefit from a clearly defined subsection that explicitly outlines the study's contributions to the field, particularly in relation to digital health and personal health information privacy.

Response: Thank you for this helpful recommendation. To improve clarity and highlight the study's contribution, we have separated the final paragraph of the Discussion into a distinct subsection titled "Implications." This section explicitly outlines the study's contributions to digital health research in low-resource settings, including its role in establishing baseline evidence on PHIPC in Myanmar and informing privacy governance and digital health policy (Implications, Page 33, Lines 574-585).

6. Conclusion

The conclusions are well grounded in the study's results and discussion, representing a key strength of the manuscript. The authors also provide clear and relevant recommendations for stakeholders and outline directions for future research.

Summary of Minor comments:

- Improve alignment and consistency between the abstract and the Objectives section, ensuring the main objective stated in the abstract is clearly reflected as the primary objective.
- Use consistent terminology throughout the manuscript (e.g., avoid interchangeable use of "extent" and "level" of privacy concerns).
- If primary and secondary objectives are intended, clearly separate and state them concisely.
- In the Study Design section, avoid restating the objective; instead, briefly justify why a cross-sectional online survey is appropriate for achieving the study aim.
- Reorganize the Methods section for clarity by separating setting, data collection, recruitment, and sampling strategy into distinct subsections.
- In the Data Collection Tool/Instruments section, clearly describe the instrument validation process (e.g., CFA), rather than placing this information in the Data Analysis section.
- Relocate instrument validation details (e.g., confirmatory factor analysis) from the Data Analysis section to the Data Collection Tool/Instruments section.
- Clarify the study timeframe to avoid confusion between the data collection period and references to the broader political context.
- Specify the names of private clinics involved in participant recruitment rather than referring only to cities.
- Improve accessibility by defining abbreviations at first mention or moving the abbreviations list earlier in the manuscript.
- Add a brief, clearly defined section highlighting the study's key contributions to the field.

Response: Thank you for these constructive and well-organized suggestions. All the points summarized above have been addressed in our detailed point-by-point responses above. Also, we have clarified the description of the study timeframe in the Setting section to explicitly distinguish the March–May 2024 data collection period from the broader political context following the February 2021 military coup. (Setting, Page 10, Lines 225-228). We believe these revisions have improved the clarity, consistency, and structural organization of the manuscript.
