# Supplementary material for: Privacy concerns regarding personal health information in Myanmar: A cross-sectional survey in a least developed country
Source: PLOS Digit Health. 2026 Mar 26;5(3):e0001007. doi: 10.1371/journal.pdig.0001007 (PMC13020815; doi:10.1371/journal.pdig.0001007)
Supplement: Attachment — Submitted filename: Peer Review BY Josiane A.10.1.2026.docx [file pdig.0001007.s005.docx]

**PEER REVIEW**

**Peer reviewer information:**

**Name:** AKINGENYE Josiane

**Location:** Kigali, Rwanda

**Job Title**: Director of Grants and Research

**Affiliation**: Informed Future Generation

**Qualification**: PG in Clinical Research and Medication Writing, Associate degree in data science, and a Bachelor's in General Nursing.

**Manuscript title**: Privacy concerns regarding personal health information in Myanmar: A cross-sectional survey in a least developed country.

**Editor Summary:**

This manuscript makes a strong and timely contribution to digital health by examining personal health information privacy concerns in a low-resource setting. The manuscript is clear and informative, with well-presented results supported by organized tables and figures, and a discussion that appropriately places the findings within existing literature. The authors demonstrate strong transparency by clearly reporting study limitations and providing practical recommendations for relevant stakeholders. In addition, the manuscript aligns well with open science expectations by indicating how and where the underlying data and tables can be accessed.

The comments raised are largely minor and focus on improving clarity, consistency, and structural organization rather than substantive methodological flaws. Key areas for improvement include clearer alignment between the abstract and objectives, consistent use of terminology, and minor reorganization of the Methods section to improve readability (e.g., clearer separation of study design, setting, recruitment, sampling strategy, and instrument validation). Additional minor clarifications related to the placement of abbreviations, study timeframe, and presentation of study contributions would further strengthen the manuscript. The authors are also encouraged to ensure that ethical approval details are clearly documented, including reference to supporting approval information.

These issues are readily addressable through revision and do not detract from the overall quality or contribution of the work. With these minor revisions, the manuscript would be well-positioned for publication.

**FEEDBACK**

1. **Abstract**

The abstract provides a clear and concise summary of the manuscript, effectively presenting the research problem, study objective, methodology, key findings, and conclusions within a limited word count. The authors have adhered to the journal’s abstract length requirement, with a total of 282 words, which is appropriate. Importantly, the abstract is well aligned with the content of the full manuscript, accurately reflecting the study’s scope and main contributions.

2. **Background and introduction**

I appreciate the clarity and accessibility with which the problem is presented in the Background and Introduction. The rationale for why this topic matters is clearly articulated, allowing readers to easily grasp the importance of personal health information (PHI) privacy. The writing is coherent, concise, and logically structured, with a smooth flow of ideas and effective comparisons drawn from related research conducted in Europe (Ireland), Oceania (New Zealand), Asia, Africa, and the United States. These comparisons help illustrate the magnitude of privacy concerns surrounding personal health information (PHI) and demonstrate how the topic adds knowledge and contributes meaningfully to digital health research.

In addition, the authors use clear and non-technical language, avoiding unnecessary jargon, which makes the manuscript accessible not only to medical professionals but also to a broader readership. This is a notable strength of the paper. Furthermore, each key idea is appropriately supported by references, demonstrating a thorough engagement with relevant literature and strengthening the credibility and scholarly rigor of the manuscript.

3. **Study Objectives**

While the Objectives section identifies the primary goal of the study, there is an inconsistency in the terminology used to describe it. The manuscript alternates between terms such as “extent” and “level” of personal health information privacy concerns (PHIPC). To avoid ambiguity, the authors should select one term and use it consistently across the abstract, objectives, and throughout the manuscript.

In addition, the Objectives section includes extensive narrative explanations and contextual framing (e.g., references to differences between developed and developing countries and political instability). While relevant, these explanations reduce the clarity of the primary objective. Such contextual information would be more appropriately placed in the Background or Discussion sections.

To strengthen coherence, the authors are encouraged to present the main objective concisely and in direct alignment with the abstract, retaining the action verb “assess not this study aimed to investigate PHIPC….” If both primary and secondary objectives are intended, these should be clearly separated and simply stated, with the objective described in the abstract presented as the primary objective and any additional aims framed as secondary.

4. **Methodology:**
a. **Study design:** The cross-sectional study design is appropriate for the study objectives. However, in the Study Design section, the authors restate the study objective (i.e., assessing the extent of PHI privacy concerns and identifying associated factors) rather than briefly explaining how the study design is suitable for achieving this aim. The authors may consider concisely justifying the choice of a cross-sectional design (e.g., its suitability for measuring the prevalence and associated factors of PHIPC at a single point in time) instead of reiterating the objective.

Please consider naming the private clinics where participants with low digital literacy were recruited, rather than referring only to the cities, to improve clarity.

b. **Setting:** In the Setting section, information related to the study setting, data collection process, and participant recruitment is combined. For improved clarity and structure, the authors may consider separating these elements into distinct subsections (e.g., Setting, Data Collection, and Recruitment).
c. **Participants:** The use of convenience sampling is understandable given the political instability in the country and the reliance on online data collection to facilitate participation. However, to improve clarity, the authors are encouraged to separate the descriptions of participants’ eligibility criteria, recruitment process, and sampling strategy into distinct subsections.
d. **Data Collection Tool/Instruments:** The assessment section provides a clear and detailed description of the data collection tools. However, the description of tool validation using confirmatory factor analysis (CFA) is currently placed under the data analysis section. The authors are encouraged to move this description to the Data Collection Tool/Instruments section, as it more appropriately reflects instrument validation.
e. **Translation process/data collection process:** procedures are clearly and well described. The manuscript provides a transparent explanation of how information was translated between the local language and English, and the data validation and quality control measures appear to be robust.
f. **Data Analysis:** The data analysis section is well described and easy to follow. However, the statement describing the validation of the questionnaire using confirmatory factor analysis (CFA) is not essential in this section and would be more appropriately placed under the Data Collection Tool/Instruments section.

**NB:** **Responses from Google Forms were exported as CSV files and analyzed using RStudio (version 2024.12.0+467).** Could be a good introductory sentence in data analysis.

g. **Ethical Approval**: This section was well stated, and this paper fulfilled ethical requirements and protected participants' privacy and protection.
5. **Results, Discussions, Evidence, and Conclusions:**

The Results section is clearly presented, with well-organized tables and figures that appropriately address the study objectives. The findings are logically arranged and easy to follow.

The Discussion section is coherent and thoughtfully written, with effective comparisons to related studies. These comparisons clearly highlight the significance of the findings, as well as similarities and differences with previous research.

I also appreciate the transparent and comprehensive reporting of study limitations. The authors clearly acknowledge key limitations related to the study design, sample size, subgroup imbalances resulting from online data collection, and the underrepresentation of rural populations (given that approximately 70% of the country is rural, while only about 25% of participants were included). This level of transparency is valuable for readers and future researchers in identifying gaps and areas for improvement.

As a suggestion, the manuscript would benefit from a clearly defined subsection that explicitly outlines the study’s contributions to the field, particularly in relation to digital health and personal health information privacy.

6. **Conclusion**

The conclusions are well grounded in the study’s results and discussion, representing a key strength of the manuscript. The authors also provide clear and relevant recommendations for stakeholders and outline directions for future research.

**Summary of Minor comments:**

- Improve alignment and consistency between the abstract and the Objectives section, ensuring the main objective stated in the abstract is clearly reflected as the primary objective.
- Use consistent terminology throughout the manuscript (e.g., avoid interchangeable use of “extent” and “level” of privacy concerns).
- If primary and secondary objectives are intended, clearly separate and state them concisely.
- In the Study Design section, avoid restating the objective; instead, briefly justify why a cross-sectional online survey is appropriate for achieving the study aim.
- Reorganize the Methods section for clarity by separating setting, data collection, recruitment, and sampling strategy into distinct subsections.
- In the Data Collection Tool/Instruments section, clearly describe the instrument validation process (e.g., CFA), rather than placing this information in the Data Analysis section.
- Relocate instrument validation details (e.g., confirmatory factor analysis) from the Data Analysis section to the Data Collection Tool/Instruments section.
- Clarify the study timeframe to avoid confusion between the data collection period and references to the broader political context.
- Specify the names of private clinics involved in participant recruitment rather than referring only to cities.
- Improve accessibility by defining abbreviations at first mention or moving the abbreviations list earlier in the manuscript.
- Add a brief, clearly defined section highlighting the study’s key contributions to the field.
